# Are Therapies That Target α-Synuclein Effective at Halting Parkinson’s Disease Progression? A Systematic Review

**DOI:** 10.3390/ijms241311022

**Published:** 2023-07-03

**Authors:** Abbie T. Rodger, Maryam ALNasser, Wayne G. Carter

**Affiliations:** 1 School of Medicine, University of Nottingham, Royal Derby Hospital Centre, Derby DE22 3DT, UK; abbie.rodger@btinternet.com (A.T.R.); mbxmna@exmail.nottingham.ac.uk (M.A.); 2 Department of Biological Sciences, College of Science, King Faisal University, P.O. Box 400, Al-Ahsa 31982, Saudi Arabia

**Keywords:** α-synuclein, α-synuclein aggregation, anti-α-synuclein immunotherapy, neurodegeneration, Parkinson’s disease

## Abstract

There are currently no pharmacological treatments available that completely halt or reverse the progression of Parkinson’s Disease (PD). Hence, there is an unmet need for neuroprotective therapies. Lewy bodies are a neuropathological hallmark of PD and contain aggregated α-synuclein (α-syn) which is thought to be neurotoxic and therefore a suitable target for therapeutic interventions. To investigate this further, a systematic review was undertaken to evaluate whether anti-α-syn therapies are effective at preventing PD progression in preclinical in vivo models of PD and via current human clinical trials. An electronic literature search was performed using MEDLINE and EMBASE (Ovid), PubMed, the Web of Science Core Collection, and Cochrane databases to collate clinical evidence that investigated the targeting of α-syn. Novel preclinical anti-α-syn therapeutics provided a significant reduction of α-syn aggregations. Biochemical and immunohistochemical analysis of rodent brain tissue demonstrated that treatments reduced α-syn-associated pathology and rescued dopaminergic neuronal loss. Some of the clinical studies did not provide endpoints since they had not yet been completed or were terminated before completion. Completed clinical trials displayed significant tolerability and efficacy at reducing α-syn in patients with PD with minimal adverse effects. Collectively, this review highlights the capacity of anti-α-syn therapies to reduce the accumulation of α-syn in both preclinical and clinical trials. Hence, there is potential and optimism to target α-syn with further clinical trials to restrict dopaminergic neuronal loss and PD progression and/or provide prophylactic protection to avoid the onset of α-syn-induced PD.

## 1. Introduction

Current demographic trends reveal that the general population is aging, and by 2050, it has been predicted that approximately one in six people in the world will be over 65, compared to approximately one in 11 in 2019 [1]. With an aging population, there is an increased risk of developing neurodegenerative diseases (NDDs), including Parkinson’s Disease (PD), which is the most common neurodegenerative motor disorder affecting the elderly. Globally, the prevalence of patients diagnosed with PD has increased by 145% between 1990 and 2016, with a 161% increase in mortality over the same period [2]. In 2016, there were 6.1 million individuals living with PD [3]. Significant risk factors other than age include genetic predisposition, sex (PD is 1·4 times more frequent in men than women), and exposure to certain chemical pollutants, including pesticides and heavy metals [3,4,5].

**Figure 1:**
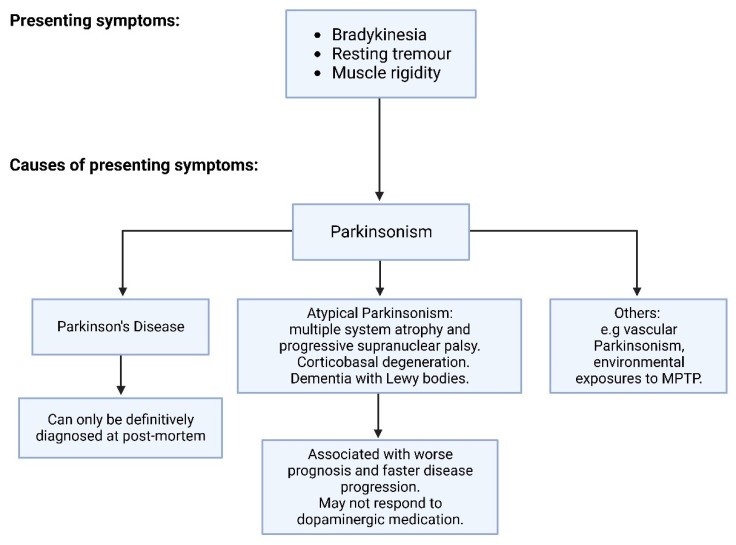
A diagram summarizing the diagnostic criteria for PD. Figure adapted from Ref. [6].

PD is characterized by the premature degradation of dopaminergic neurons in the substantia nigra pars compacta (SNpc) [4,6]. The loss of dopaminergic activity in the basal ganglia typically leads to a triad of motor symptoms: bradykinesia, resting tremor, and muscular rigidity, collectively referred to as parkinsonism [4,6]. PD is also associated with a range of non-motor symptoms, which often precede the characteristic motor symptoms for years or even decades [6]. Some of these include cognitive decline, sleep disorders, psychiatric impairment, and fatigue [4,6]. To ensure the correct and most effective treatment is provided, it must be distinguished whether the presenting parkinsonism is a result of PD rather than other underlying causes [6] (Figure 1).

Current treatments for PD are restricted to symptomatic approaches to counter the dopamine deficit through the administration of levodopa and/or dopamine receptor agonists, but these often display decreased efficacy over time and can induce undesired side effects, including dyskinesias [6,7].

### 1.1. Pathophysiology of Parkinson’s Disease

#### 1.1.1. Genetics of PD

The aetiology of PD is multifactorial, with a combination of genetic inheritance, environmental influences, and a range of physiological factors [4,5,6,8]. Idiopathic forms of the disease are the most common, with monogenic, inherited PD constituting only approximately 5–10% of all cases [5,8,9]. Monogenic forms of the disease include autosomal dominant mutations in the *SNCA*, *LRRK2,* and *VPS35* genes and autosomal recessive mutations in *PINK1*, *PRKN,* and *GBA* [5,8,9]. SNCA encodes for alpha-synuclein (α-syn), and missense mutations or multiplications in this gene result in an earlier age of onset and rapid disease progression, thereby supporting the posit that abnormal α-syn production and/or activity can play a critical role in PD pathogenesis [8,9,10].

#### 1.1.2. Neuroanatomy of PD

The premature degradation of dopaminergic neurons is most prominent in the ventrolateral tier of the SNpc, with neurons that project directly to the dorsal putamen of the striatum [4]. Nigrostriatal dopaminergic cell loss leads to a gradient of dopamine depletion, causing an imbalance between the theoretical direct and indirect movement-related pathways within the basal ganglia, resulting in parkinsonism features [4,5,6,11]. Specifically, dopaminergic innervation has an inhibitory effect on the thalamus and therefore affects movement [11]. In a healthy subject, dopaminergic innervation activates the direct pathway and inhibits the indirect pathway to decrease stimuli to the globus pallidus pars interna (GPi) [11]. In the PD patient, the loss of SNpc dopaminergic innervation increases activity in the indirect pathway and decreases activity in the direct pathway [11]. This results in excessive GPi output and, subsequently, over-inhibition of the thalamus and the cortex, resulting in the typical parkinsonism features [11]. Cell loss can also be found in non-dopaminergic neurotransmitter processes, such as those supporting GABAergic and cholinergic systems [6,11]. Neurodegeneration in these systems may account for early pre-motor symptoms and will not be improved solely by dopamine replacement therapies [6,11].

### 1.2. α-Syn Toxicity in PD

**Figure 2:**
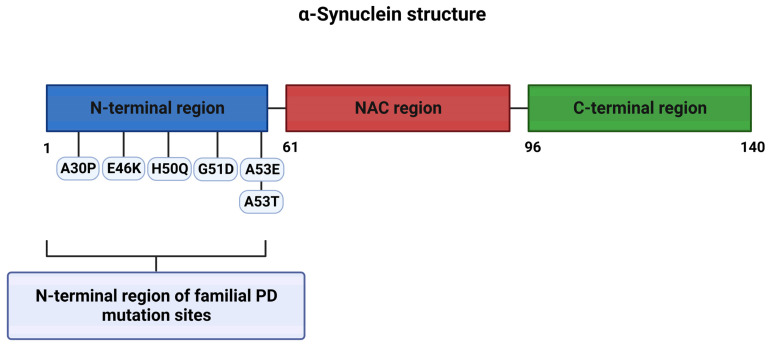
The structure of α-synuclein and positions of the familial PD mutations.

α-syn is a soluble protein of 140 amino acids with a predicted molecular weight of ≈14,000 Da that can be divided into three regions: an amino-terminal lipid-binding region, a central hydrophobic non-amyloid-β region (the non-amyloid-β component (NAC) domain), and a disordered carboxy-terminal (Figure 1). Autosomal dominant forms of familial PD arise from N-terminal point mutations in α-syn, and increased copy numbers (duplications or triplications) of the SNCA gene [8,9,10,12,13] (Figure 2).

A complete dissection of the physiological function of α-syn has not yet been completed, but its relatively high expression levels in nervous tissue and particularly localization to presynaptic nerve terminals indicate a role in synaptic activity, vesicle trafficking, and control of dopamine release [12,13,14,15]. The influence and importance of α-synuclein functionality on post-synaptic membranes have also been documented, including effects on glutaminergic neurotransmission [14]. Furthermore, the presence of α-syn in other subcellular compartments, including the nucleus, mitochondria, endoplasmic reticulum, and endolysosomal system, hints at multiple cellular functions and protein interactions, and these have not yet been fully delineated [15].

**Figure 3:**
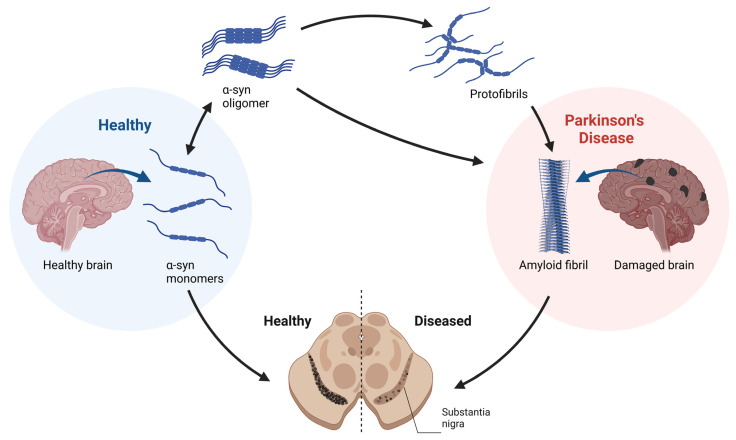
A summary of the α-syn aggregation process and potential contribution to PD. α-syn can exist as naturally occurring monomers or other oligomeric forms such as tetramers, but with the appropriate trigger(s), it can aggregate to form neurotoxic oligomers and fibrils within Lewy bodies. Pathological examination of the brain in PD patients displays a pigment reduction within the substantia nigra because of dopaminergic neuronal degeneration.

α-syn can shift between multiple protein conformations due to its structural flexibility, moving from monomers to oligomers (soluble forms) such as tetramers [16,17] and then to potentially toxic proteinase-k-resistant oligomers and fibrils (Figure 3) [12,13,14]. α-syn also undergoes extensive protein post-translational modifications (PTMs), including phosphorylation, acetylation, and isoaspartate formation, PTMs that may influence protein aggregation and toxic potential [17,18].

α-syn oligomers and fibrils may both be neurotoxic, and some studies suggest that oligomerization leads to particularly toxic species that can induce redox stress and a loss of axonal function [14,19,20,21,22].

The pathological hallmark of PD is the presence of LBs, and these contain a heterogenous mixture of molecules, including α-syn and proteins involved in mitochondrial function, autophagy, and the ubiquitin-proteasome system (UPS) [23]. Lewy pathology is hypothesized to progress in a pre-determined sequence over the course of PD for some patients, with six stages of Lewy pathology that start in the peripheral nervous system (PNS) (stage 1 within the dorsal motor nucleus of the glossopharyngeal and vagal nerves and anterior olfactory nucleus) and develop into the limbic system (for stages 5 and 6, LBs are present within the neocortex) [24]. The potential spread of LB pathology provides a rationale for the presentation of early (prodromal) non-motor PD symptoms and, subsequently, motor symptoms associated with dopaminergic neuronal loss that may arise through cell-to-cell (prion-like) α-syn spread between anatomically connected brain regions, possibly originating in the gut and then spreading to the brain via the vagal nerve [24,25,26].

### 1.3. α-Synuclein as a PD Therapeutic Target

The complexity of PD is compounded by genetic and environmental influences, and this renders it difficult to determine a universal pathophysiological mechanism that results in dopaminergic neuronal loss. With the current lack of neuroprotective or neurorestorative therapies for PD, targeting α-syn toxicity is a strategy that may benefit both familial and idiopathic PD.

Hence, this review aimed to assess the viability of α-syn as a therapeutic target for PD. Potential anti-α-syn mechanisms include suppression of α-syn expression and aggregation; enhancement of α-syn degradation; and prevention of α-syn spread [27,28]. This systematic review will collate and critically appraise the experimental literature that has considered targeting α-syn as a therapy in animal models and human studies of PD. Clinical data and study outcomes will be assessed to determine whether α-syn is a viable target to limit the pathogenesis and/or progression of PD.

## 2. Materials and Methods

A systematic review of the literature was carried out according to the Preferred Reporting Items for Systematic Reviews and Meta-Analyses (PRISMA) [29].

### 2.1. Information Sources and Search Strategy

An electronic literature search was performed using MEDLINE and EMBASE (Ovid), PubMed, the Web of Science Core Collection, and Cochrane databases to collate clinical evidence investigating the use of α-syn as a target for PD therapy. A combination of the Boolean operators (AND/OR) and a range of field tags were recruited for the following major search terms: Parkinson’s disease, Parkinson*, anti-alpha-syn*, target* alpha-syn*, decreasing alpha-syn*, reducing alpha-syn*, in vivo, pre-clinical, clinical, human, animal, primate, monkey, rodent, mouse, mice, rat (refer to Supplementary Data S1 for details of the full search strategy). 

Additional hand-searched articles were located in appropriate bibliographies and review papers. The full OVID, PubMed, and Cochrane search strategy is provided in Supplementary Data S1. The provisional filters applied, when possible, were the year of publication (between 2012 and 2022), type of publication (removing reviews), and English-language publications.

### 2.2. Eligibility Criteria

All search results were exported to the reference manager EndNote for text analysis and the removal of publications that did not fit the pre-defined eligibility criteria or were duplicates. Articles included in the review were full-text publications published in the English language from January 2012 to December 2022 that investigated the use of α-syn as a therapeutic target in human or animal models in vivo without constraints on study outcomes or intervention type. 

The accepted studies investigated targeting α-syn specifically as a novel therapeutic technique in PD models, excluding other studies that indirectly influenced α-syn levels and other neurodegenerative diseases that exhibit LB pathology, such as Lewy body dementia (LBD). Repurposed drugs were not included in the review in order to specifically focus on novel molecular entities and new strategies that target α-syn. Studies were excluded if they were not performed on rodents, primates, or human subjects, if the publication was not in the English language, or if they were review articles or conference abstracts. Publications ≥11 years were excluded to provide a contemporary review of this therapeutic area.

### 2.3. Study Selection and Data Collection Process

**Figure 4:**
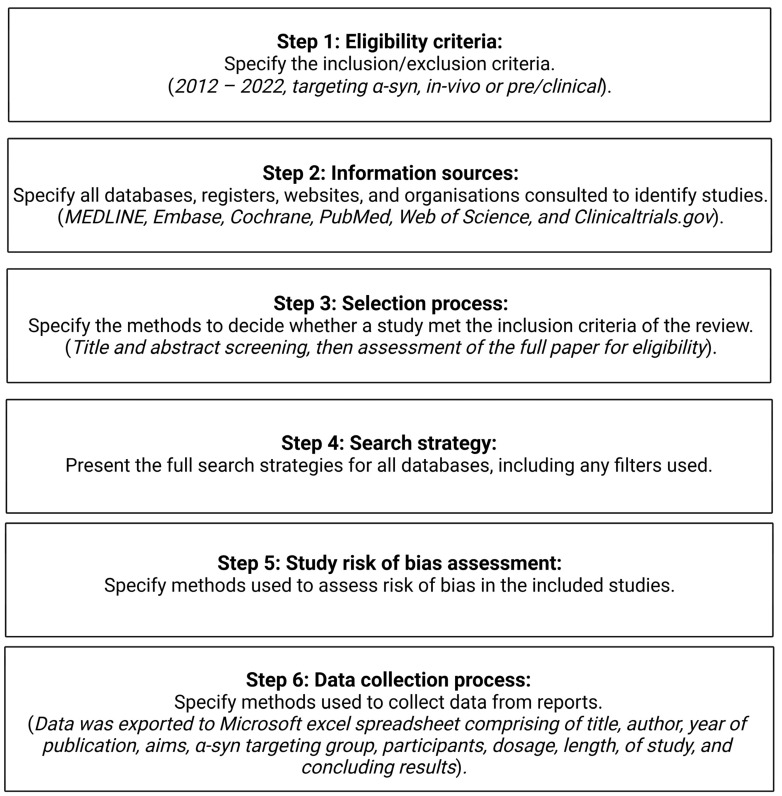
A summary of the systematic review stages as adapted from the PRISMA 2020 statement: an updated guideline of reporting systematic reviews [29].

Identified studies were assessed for eligibility, and duplicate publications were removed. Eligible publications were retrieved using the University of Nottingham Library (NUsearch). These publications were exported to a Microsoft Excel data spreadsheet with the following variables extracted: title, author(s), year of publication, the aim of the study, α-syn target group, intervention, population, dosage, length of study, and study results. Due to the mixed quantitative and qualitative nature of the study outcomes, a meta-analysis was not performed. The ‘SYRCLE’s risk of bias tool for animal studies was used to consider the risk of bias from the methodologies collated [30]. A summary of the methods section, in coordination with the PRISMA checklist, is included in Figure 4 [29]. Selected papers were checked and validated by the manuscript authors. Figures were produced using BioRender (https://www.biorender.com/).

## 3. Results

**Figure 5:**
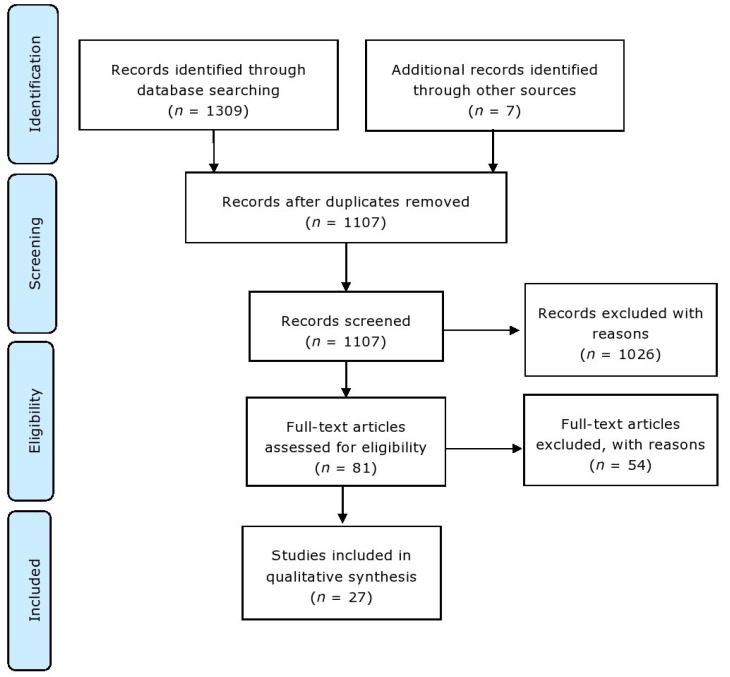
Preferred reporting items for systematic reviews and meta-analysis (PRISMA) flow chart displaying the study identification and selection process [29].

A total of 1309 articles were identified from the preliminary database search, and an additional 7 articles were identified from hand-searching key papers and reviews. Duplicates were removed to create a total of 1107 articles, which were screened via title and abstract. Another 1026 articles were removed, leading to 81 papers for full-text assessment (Figure 5). Out of these 81 studies, 54 did not meet the pre-defined eligibility criteria and were rejected based on the following: they were performed in vitro, lacked specificity and therapeutic focus, investigated repurposed drugs, or were performed on other neurodegenerative disease models. The remaining 27 articles fulfilled the eligibility criteria and were included in the analysis. The majority focused on immunological techniques that targeted α-syn (*n* = 10), gene therapy approaches to reduce SNCA expression (*n* = 7), or inhibition of α-syn aggregation and/or promoting α-syn degradation (*n* = 10). A flow chart was generated according to the preferred reporting items for systematic reviews and meta-analysis (PRISMA) that displayed the study identification and selection process [29] (Figure 5), and studies were considered for risk of bias [30].

### 3.1. Background Mechanisms

#### 3.1.1. Immunotherapeutic Interventions 

Aggregated α-syn represents a possible target for immunotherapeutic modalities due to its proposed toxicity and causative link to PD pathology. Immunotherapies that target α-syn could reduce α-syn aggregation, inhibit prion-like spreading and neuroinflammation, or promote intracellular or extracellular clearance [31]. One clinical trial was terminated since it did not reach its primary outcomes [32,33], and one study was ongoing at the time of this review [34]. The treatment strategies and study objectives of the immunotherapeutic interventions targeting α-syn have been categorized into clinical and preclinical (in vivo) studies and placed in ascending order of publication in Table 1.

#### 3.1.2. Therapeutic Interventions That Target the SNCA Gene

The protein product of the SNCA gene, α-syn, can potentially self-associate into toxic oligomers. Hence, reducing α-syn protein production and/or aggregation at the source via gene-silencing mechanisms is a potentially fruitful neuroprotective and/or PD therapy option. Of the seven studies targeting SNCA expression (*n* = 7); six focused on non-viral gene therapy delivery, including antisense or heteroduplex oligonucleotides (ASOs/HDOs), nanoparticles, and novel peptides, and one study utilized rabies virus glycoprotein (RVG) to deliver RNA interference (RNAi) [46,47,48,49,50,51,52]. The treatment strategy and study objectives for the gene therapies targeting SNCA have been categorized into non-viral or viral delivery and placed in ascending order of publication in Table 2.

#### 3.1.3. Interventions That Target the Reduction of α-Syn Aggregates

Inhibiting α-syn aggregation is an attractive target for combating α-syn-induced PD, and several groups have focused on the disaggregation of α-syn as a therapeutic mechanism [53,54,55,56,57,58,59,60,61,62], and these have been summarized and placed in ascending year of publication in Table 3.

### 3.2. Study Characteristics

Of the 28 studies included, the majority utilized rodent models: one included cynomolgus monkeys [40], and the remainder were clinical trials (from Phase I to Phase II). Immunotherapeutic interventions targeting α-syn were either active immunization or passive mechanisms typically involving humanized monoclonal antibodies/nanobodies, with immunization via subcutaneous [35,41,42], stereotaxic injection [36,40,43], intravenous [37,38,40,45] intraperitoneal [39], and intradermal [44] routes. Studies targeting SNCA expression (*n* = 7) focused on the use of oligonucleotides or novel transport molecules to target SNCA expression [46,47,48,49,50,51,52]. Studies were further divided into viral and non-viral methods of delivery. Studies that explored inhibition of the aggregation of α-syn (*n* = 10) used a range of interventions, including aggregation inhibitors, targeted degradation and clearance, and molecular tweezer inhibitors [53,54,55,56,57,58,59,60,61,62].

### 3.3. Biochemical and Immunohistochemical Outcomes

Changes in α-syn, often in aggregated form, dopamine, and tyrosine hydroxylase (TH) levels were the end-point measurements used to gauge the effectiveness of therapeutic interventions. This is predicated upon the concept that the level of aggregated α-syn is a contributing factor to neurotoxicity and associated dopaminergic neuronal loss in this form of PD. TH is the enzyme that catalyzes the hydroxylation of tyrosine to L-DOPA, the precursor to dopamine, and therefore provides a surrogate for dopamine levels [63].

#### 3.3.1. Immunotherapeutic Outcomes

Vaccination against α-syn delayed striatal pathology and reduced striatal α-syn aggregation after eight weeks (*p* < 0.05) [35]. This schedule of vaccination induced the infiltration of B and T-cells and the production of antibodies to target α-syn in rats overexpressing human α-syn [35].

The human nanobody constructs VH14 and NbSyn87, when linked to a proteosome-targeting PEST sequence, induced a significant reduction of serine 129-phosphorylated (aggregated) α-syn (*p* < 0.05) [36]. Improvements of nigrostriatal dopaminergic tone were more extensive with VH14*PEST than NbSyn87*PEST, with the former agent producing a 49% increase in TH-labelled cells in the SN compared to control animals (*p* < 0.01), 28% increased dopamine transporter expression (*p* < 0.05), and median dopamine concentration increased by approximately 3-fold higher in the treatment group (*p* = 0.13) [36].

PRX002, a humanized IgG monoclonal antibody, was designed to target the C-terminus of α-syn and inhibit neuron-to-neuron transfer of α-syn in patients with PD [37]. Following a third PRX002 dose of 3, 10, 30, or 60 mg/kg, there was a significant and dose-dependent reduction in serum free-to-total α-syn (*p* < 0.001) [37]. 

A novel DNA aptamer, packaged into RVG-exosomes, significantly reduced insoluble α-syn aggregates (*p* < 0.01) in a preformed fibril (PFF) model and was able to rescue mouse grip strength 30 days post-treatment [39]. 

A pre-clinical assessment of the functionality of MEDI1341, a high-affinity antibody directed to the C-terminus of α-syn, showed attenuated hippocampal and neocortical α-syn levels (*p* < 0.001) [40].

PD01A, an AFFIRS peptide vaccine produced an IgG antibody response to α-syn, and a mean reduction of 51% of oligomeric α-syn in cerebrospinal fluid (CSF) [41]. These findings supported an ongoing phase II clinical trial, which is due to finish in 2026.

Similarly, PD03A, a short 10 amino-acid synthetic peptide that acts as a molecular mimic of an epitope in the C-terminal of α-syn was evaluated using a Phase I clinical study that characterized its safety and tolerability, and reported that 88% of immunized patients displayed immunological responses towards PD03A with significant antibody titres at 40 weeks after administration of 15 µg (*p* = 0.0258) or 75 µg (*p* = 0.0175) of the peptide [42]. 

PFFNB2 was another nanobody-based intervention that was able to recognize α-syn preformed fibrils (PFFs) rather than α-syn monomers. The coupling of PFFNB2 with an adeno-associated virus (AAV)-encoding an enhanced green fluorescent protein (AAV-EGFP-PFFNB2) resulted in a nanobody able to reduce aggregated (phospho-Ser129 positive) α-syn (*p* < 0.001) and limit the pathological spread of α-syn fibrils [43]. 

A novel ‘Win the Skin Immune System Trick’ (WISIT) vaccine was developed to target skin-resident dendritic cells to induce substantial B and T cell responses [44]. The WISIT vaccine candidate type-1 (CW-type 1) was able to significantly reduce the level of aggregated (phospho-Ser129 positive) α-syn across all brain regions examined (*p* < 0.05) [44].

A modified bispecific antibody, RmAbSynO2-scFv8D3, was engineered to target aggregated α-syn as well as the transferrin receptor for facilitated brain uptake across the BBB. Application of the bispecific antibody was able to reduce levels of α-syn aggregates in the cortex (*p* < 0.05) and midbrain (*p* < 0.005) in transgenic mice [45]. 

Immunological interventions targeting α-syn have been listed in ascending order of publication in Table 4.

#### 3.3.2. Gene-Therapy Outcomes

A range of delivery methods have been exploited to target the SNCA gene and reduce α-syn protein expression. Incorporation of a low molecular weight polyethyleneimine, PEI F25-LMW, facilitated the transport of RNA interference (RNAi) into Thy-1-α-syn transgenic mice, and this produced a knockdown of SNCA mRNA of up to 67% (*p* = 0.003), with a corresponding 31% reduction of α-syn protein in the medial striatum (*p* = 0.018) [46]. 

The conjugation of short interfering RNA (siRNA) or antisense oligonucleotide (ASO) molecules with indatraline (IND) was utilized to reduce α-syn expression in mouse neurons. SNCA mRNA levels were significantly lowered one-day post-treatment with either IND-499-siRNA or IND-1233-ASO administration (*p* < 0.01), with an associated reduction in α-syn protein (*p* < 0.05) [47]. IND-1233-ASO also enhanced forebrain dopaminergic release in response to veratridine (*p* < 0.05) [47].

A viral exosome, rabies virus glycoprotein (RVG), was utilized to transport short hairpin RNA mini circles (shRNA-MCs) constructs into the CNS of a PD animal model [48]. A 90 day treatment with shRNA-MCs significantly decreased α-syn levels (*p* = 0.033) and combated some of the loss of dopaminergic neurons in transgenic mice, with a significant increase in TH-immunoreactivity (*p* = 0.028) [48]. 

The transportation of a siRNA (to target α-syn) across the BBB was successful via coupling to an 11 amino acid sequence from the apoB protein (ApoB^11^) and a 9 amino acid arginine linker [49]. Delivery of the ApoB^11^/si α-syn significantly reduced α-syn protein levels (*p* < 0.05) and partially restored the loss of TH-positive dopaminergic loss in the striatum (*p* < 0.05) [49]. 

Delivery of an antisense oligonucleotide, ASO1, suppressed SNCA mRNA expression with a dose-responsive reduction in α-syn aggregates in a PFF model of PD and normalized striatal dopamine levels [50]. Tat-βsyn-degron, a novel α-syn knockdown peptide that promotes proteasomal degradation of α-syn, significantly reduced α-syn aggregations in the SN of mice (*p* < 0.01) and protected against 1-methyl-4-phenyl-1,2,3,6-tetrahydropyridine (MPTP)-induced parkinsonian (dopaminergic) neuronal damage [51]. 

Use of an α-syn heteroduplex oligonucleotide (α-syn-HDO) attenuated SNCA expression and α-syn protein production in the SN (*p* < 0.05), ameliorated dopaminergic neuron degeneration (*p* < 0.01), and improved motor performance in transgenic mice; effects linked to induction of brain-derived neurotrophic factor (BDNF) [52]. 

A summary of the biochemical and immunohistochemical outcomes from studies targeting α-syn via gene therapy has been included in Table 5 and listed in ascending year of publication.

#### 3.3.3. Outcomes from Agents That Reduce the Levels of α-Syn Aggregates

Anle138b, a novel oligomer modulator, blocked the formation of oligomeric α-syn (*p* < 0.001) and ameliorated the loss of motor performance (by rotarod assay) for a rotenone-induced model of PD and A30P-α-syn transgenic mice (*p* < 0.01) [53]. 

Overexpression of the neuronally expressed developmentally down-regulated gene 4 (Nedd4), a ubiquitin ligase that targets substrates including α-syn to endosomal-lysosomal degradation, significantly reduced α-syn levels and was able to partially restore an α-syn-induced loss of TH-labelled neurons (*p* < 0.05) [54]. 

Treatment of A30P transgenic mice with a prolyl oligopeptidase (KYP-2047) reduced oligomeric α-syn and increased striatal dopamine levels after a 28 day treatment (*p* = 0.01) [55]. 

A therapeutic strategy for reducing α-syn toxicity by displacing α-syn from the membrane utilized NPT100-18A [53]. This compound interacted with a domain on the α-syn C-terminus and significantly reduced the formation of proteinase K-resistant α-syn aggregates (*p* < 0.05) and reduced neurodegenerative pathology in mThy1-α-syn transgenic mice [56]. 

Graphene quantum dots (GQDs) inhibited α-syn fibril formation (*p* = 0.001), alleviated motor deficits, and protected against α-syn PFF-induced loss of dopaminergic neurones (*p* = 0.0282) [57].

The molecular tweezer, CLR01, binds to lysine residues considered critical to the oligomerization of α-syn [55]. CLR01 reduced α-syn aggregation in vitro, significantly improved motor deficits, and reduced oligomeric α-syn (*p* = 0.0286) in transgenic mice that overexpress α-syn [58]. 

The small molecule β-carboline alkaloid harmol acts as an autophagy enhancer, and administration in vivo reduced α-syn levels in the substantia nigra and prefrontal cortex (*p* < 0.05) and improved motor deficits in PD transgenic mice [59].

Acidic nanoparticles (aNPs) can promote lysosomal degradation of α-syn and reduced α-syn pathology to protect nigral dopaminergic neurons (*p* < 0.05) in a PD model [60].

The administration of the small molecule *trans*-4′-acetyl-3′-tigloylkhellactone (racemic peucedanocoumarin IV (PCiv)) provided an agent able to suppress α-syn aggregation (*p* < 0.01) and partially restore dopaminergic neuron loss (*p* < 0.001) and motor function in a transgenic model of PD [61].

A rabies viral polypeptide (of 29 amino acids) modified red blood cell membrane (RBCm) was used to encapsulate curcumin nanocrystals (RVG29-RBCm/Cur-NCs), and this nanodecoy delivery of curcumin was able to inhibit α-syn aggregation, restore the number of TH-positive neurons, and attenuate motor deficits in a mouse model of PD [62].

A summary of the biochemical and immunohistochemical analyses in PD groups compared to interventions targeting α-syn aggregates has been included in Table 6, with studies listed in ascending order of publication.

## 4. Discussion

The complex and multifaceted pathology of PD renders it a challenging problem to devise a broad or universal treatment to prevent disease progression. Although LB pathology is not the only causative factor for PD, it is known that in the presence of α-syn oligomers and fibrils, neurodegeneration occurs [4]. Therefore, treatments that target α-syn oligomeric and/or fibrillar species that are toxic to neurons may reduce neurodegeneration, but this therapeutic strategy has yet to be fully translated into efficacious clinical studies. Certainly, α-syn aggregation may not be the sole cause of dopaminergic neuronal loss, and the complex interactions between the aggregates of pathogenic proteins and neurodegeneration continue to evolve. However, the effect of a therapy on oligomeric and fibrillar α-syn and dopaminergic neuronal activity are the primary indicators of efficacy against PD-related neurodegeneration. After collating qualitative and quantitative data in this review, it can be concluded that α-syn is a viable target to limit α-syn-induced PD. The significant reduction in α-syn aggregations and the corresponding increase in dopaminergic innervation observed from in vivo (animal) studies indicate a clear benefit of targeting α-syn in preventing the progression of α-syn-induced PD. Similarly, the positive outcomes from the clinical studies assessing the efficacy and safety of immunotherapies provide evidence of the potential viability of anti-α-syn therapies. 

### 4.1. Mechanisms of Immunotherapeutic Interventions Targeting α-Syn

The clinical efficacy and utilization of a broad number of immunotherapy and immunomodulatory drugs for the treatment of multiple sclerosis [64] have encouraged optimism for the adoption of similar treatment strategies for other neurodegenerative diseases. For α-syn-induced PD, this has involved both active and passive immunotherapeutic strategies. 

#### 4.1.1. Active Immunotherapy

Active immunotherapeutic techniques have been adopted to stimulate humoral and cellular responses to provide long-term α-syn clearance by directing the host’s immune response to target α-syn. The advantages of active immunization include the potential for the generation of polyclonal responses and an approach that will obviate the need for regular (passive) dosing, which may be costly to implement. However, a strategy is needed that will induce a suitable adaptive response without the loss of tolerance or the induction of chronic and damaging neuroinflammation. Vaccination using full-length human recombinant α-syn was able to reduce the levels of aggregated α-syn in a model of Lewy body dementia (LBD) [65]. Subsequently, a protective vaccination strategy that potentiated natural tolerance against human recombinant α-syn reduced the neuroinflammatory response in a PD model [35]. The induction of microglia (CD4^+^/MHC II cells) towards α-syn pathology also produced a Treg response and IgG deposition [35]. 

For human clinical studies, Phase I clinical trials have explored the safety and efficacy of two anti-α-syn vaccines: PD01A and PD03A [41,42]. These vaccines consist of a short antigenic peptide used to impersonate an epitope in the native C-terminal region of human α-syn [42]. Both peptides were coupled with the carrier protein keyhole limpet haemocyanin and an adjuvant of aluminium hydroxide to provide T-cells with the required epitope to direct the production of antigen-specific antibodies from plasma cells [42]. The antigenic peptides were designed to remove α-syn immune tolerance by activating B-cell recruitment and producing high antibody titres to the immunizing peptide without inducing an auto-immune response [41,42]. 

Other active immunization studies include the use of UB-312, a synthetic peptide-based vaccine utilized for the first in-human randomized, controlled trial (NCT04075318) to assess its safety and efficacy [34]. In the pre-clinical assessment of UB-312 using an α-syn overexpression PD mouse model (Thy1SNCA/15), the level of α-syn oligomeric structures was significantly reduced, improved motor performance was observed, and there was no sustained neuroinflammatory response [66]. 

An alternative active vaccination was adopted through the generation of a peptide vaccine platform for the production of a WISIT (win the skin immune system trick) vaccine with a β-glucan sequence fused with variable B and T-helper cell peptide epitopes [44]. Administration of the WISIT candidate type 1 (CW-type-1) vaccine to mice was able to significantly reduce the level of aggregated (phospho-Ser129 positive) α-syn across all brain regions examined [44].

Recent studies have also evaluated the immunogenicity of a recombinant DNA vaccine to multiple B-cell epitopes of α-syn, PV-1950R, that was able to reduce total and protein-kinase-resistant α-syn [67,68]. Hence, active immunotherapy may prove a useful means to limit toxic α-syn accumulation and the pathological consequences that arise from its neurotoxicity.

#### 4.1.2. Passive Immunotherapy

By contrast to active vaccination, passive immunotherapies may benefit from pre-determined epitope selectivity via the provision of target antigens but have the drawback of relatively short half-lives of antibody fragments and therefore the need for frequent infusions. Since a prion-like spread of α-syn may be critical to disease progression [24,25,26], humanized IgG monoclonal antibodies may be able to halt this neurotoxic cell-to-cell spread. PRX002, a high-affinity monoclonal antibody directed against the C-terminus of aggregated α-syn was well tolerated in healthy volunteers [37] and may provide a useful means to limit α-syn neurotoxic spread; in keeping with preclinical assessments of PRX002 that demonstrated the ability to block cell-to-cell transmission and ameliorate memory and learning deficits in an α-syn over-expression model (mThy1-α-syn) [69]. Similarly, MEDI134, another highly selective monoclonal antibody directed to the C-terminus of α-syn, was effective at reducing the pathological spread of α-syn in vivo [40]. 

BIIB054, a fully human-derived monoclonal antibody directed against an N-terminal epitope of α-syn, was assessed for safety, tolerability, and pharmacokinetics in a Phase I trial (NCT02459886) [70]. However, the phase II clinical trial (NCT03318523) using BIIB054 was terminated for not meeting its primary outcome measure for year 1 and secondary outcome measures [32,33]. Hence, thus far, PRX002 remains a current candidate for a clinically approved monoclonal antibody treatment for PD (NCT03100149), with an estimated completion date of September 2026 [37]. 

Another human monoclonal antibody, ABBV-0805, active against α-syn aggregates, has been assessed using preclinical PD mouse models [71]. However, ABBV-0805 did not complete a Phase I clinical trial (NCT04127695) [72] and was withdrawn, so whether ABBV-0805 will be taken forward for future human studies is yet unknown. 

#### 4.1.3. Alternative Immunotherapies 

The proteinaceous nature and molecular size of antibodies can potentially be a hindrance to their use, such that accessing wholly intracellular targets can be problematic, and antibodies themselves could potentially be immunogenic. To circumvent this and other limitations associated with monoclonal antibody production and shelf-life, antibody replacements have been developed for targeting α-syn. 

Genetically engineered nanobodies are single-domain antibody fragments derived from IgG of approximately 15 kDa that can exhibit BBB penetrance [73]. The human nanobody constructs, VH14 (directed against the NAC region of α-syn) and NbSyn87 (directed against the C-terminal region of α-syn), when coupled with a proteasome-targeting PEST sequence, provided a means to enhance the degradation and clearance of the target α-syn antigen [36]. The VH14*PEST nanobody (intrabody) also improved motor function in an α-syn-induced PD rat model [36]. 

The fusion of another nanobody, PFFNB2, to an adeno-associated virus (AAV)-encoding EGFP (AAV-EGFP-PFFNB2) facilitated the targeting of fibrillar α-syn to limit prion-like α-syn spread in transgenic mice [43]. 

Alternatively, a high affinity, low molecular weight, single chain variable fragment (scFv), (scFv-sMB08) has been used to target both oligomeric and PFF α-syn and was able to protect neurons, limit α-syn spread, and partially attenuate the loss of motor dysfunction in PD models [74]. 

Preliminary data with a modified bispecific antibody that utilized a functional variable region with an affinity for α-syn as well as one for the transferrin receptor showed that it was able to cross the BBB and reduce α-syn oligomers within the cortex and midbrain [45].

Aptamers are single-stranded oligonucleotides (DNA or RNA) that can bind to a range of targets, including proteins. In comparison to antibodies, aptamers may be advantageous for their ease of production and modification while still retaining high target affinity [75]. Two DNA aptamers that were packaged into RVG-exosomes to facilitate CNS delivery were able to target α-syn and inhibit intracellular α-syn aggregation [39]. 

#### 4.1.4. Immunotherapeutic Conclusions

**Figure 6:**
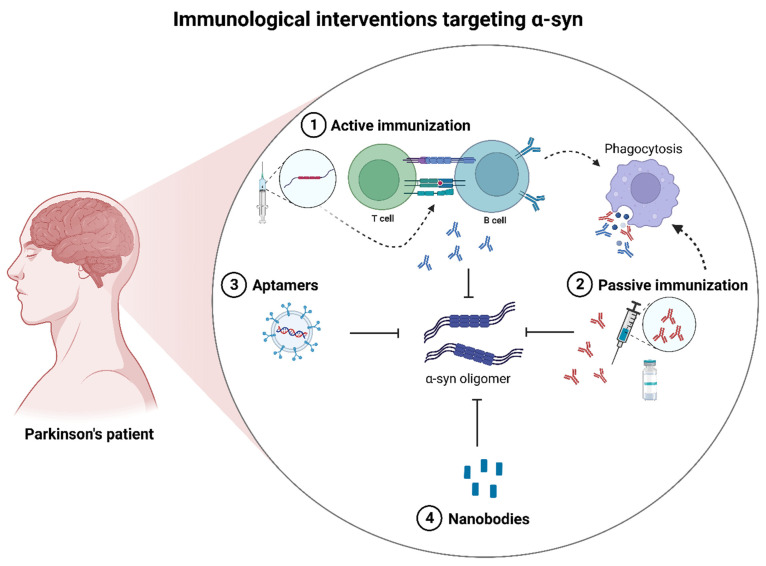
A summary of the main strategies for immunological interventions that target α-syn. Strategies employed include 1. Active immunization 2. Passive immunization 3. Aptamers, and 4. Nanobodies. Dashed arrows indicate the possible route of processing.

Although targeting AD by immunotherapeutic means has typically resulted in high-profile failures, antibody and alternative immune-based treatments for synucleinopathies may still represent viable therapeutic options [31]. Given the evidence for the prion-like behavior of α-syn [24,25,26], halting α-syn spread through targeted immunotherapy could certainly reduce disease progression. A summary of the immunotherapy mechanisms for the treatment of PD has been included in Figure 6. 

### 4.2. Mechanisms of Targeting SNCA Expression

Reducing α-syn protein levels via gene-silencing mechanisms is an attractive therapeutic approach to limit the potential production of pathogenic α-syn. However, there are a multitude of challenges to overcome when designing an appropriate gene-delivery system. These include the requirement to deliver molecules that efficiently pass across the BBB and that alter specific gene expression for a sufficient amount of time to impact the course of the disease. Adeno-associated viral (AAV) vectors can be an effective means for RNAi knockdown of a target gene; however, there may be concerns arising from vector immunogenicity [76]. Hence, there may be benefits to conjunctive therapy, in which the nucleotide used to target the gene of choice is paired with an inert molecule that can assist with transportation across the BBB.

#### Viral and Non-Viral Delivery of RNA-Based Gene Therapy

A branched polyethyleimine (PEI) of 4–12 kDa was successfully as a non-viral vector to mediate the delivery of RNAi into the brain and significantly reduce SNCA gene expression and α-syn protein levels [46]. PEIs are cationic polymers that form non-covalent complexes with the siRNA and protect it from degradation, thereby facilitating nucleotide transit and cellular take-up via endocytosis [77]. 

By conjugating an antisense oligonucleotide (ASO) or small interfering RNA (siRNA) with the triple monoamine uptake inhibitor, indatraline (IND), the IND-1233-ASO or IND-siRNA conjugates, when administered intranasally, were able to reduce the production of α-syn by targeting SNCA specifically within brainstem monoamine nuclei [47]. 

MCs are double-stranded DNA vectors that can be added to RVG-exosomes in order to deliver shRNA to knockdown SNCA expression as a PD therapy [48]. The RVG (brain-targeting) peptide on the exterior surface of the exosome provided a means for direct delivery to the brain, and the shRNA-MC constructs demonstrated the potential for relatively long-term downregulation of the α-syn (SNCA) gene for six weeks [48]. More long-term downregulation of SNCA may be clinically advantageous and obviate the need for frequent re-administration of gene dosing treatments. 

To facilitate transport across the BBB and take-up by neurons, siRNA was coupled to a novel peptide vector derived from ApoB that can bind to low-density lipoprotein (LDL) receptors on the surface of endothelial cells of the BBB and be taken up via endocytosis [49]. Although this approach significantly reduced α-syn protein levels, a concern regarding systemic delivery of peptides conjugated to siRNA is their potential transport to peripheral organs as well as the CNS [49] but given that α-syn is predominantly expressed in neurons, this may not be of detriment.

Although non-viral strategies for SNCA knockdown via ASOs have shown useful preclinical efficacy [50], a concern is a need for chronic and repeated infusions of ASOs into the brain to maintain gene knockdown. Useful BBB and plasma membrane penetrance may be provided using an α-syn binding peptide coupled with a domain for proteasomal degradation [51]. A Tat-βsyn-Degron peptide was designed, composed of three domains, a plasma layer transduction domain (Tat), an α-syn binding domain (βsyn), and a proteasomal targeting domain (degron) [51]. The effective delivery of Tat-βsyn-degron and rapid degradation of α-syn [51] are useful traits for a potential PD treatment. 

An alternative targeting of the SCNA gene is via targeted injection of heteroduplex DNA/RNA oligonucleotides (α-syn-HDO), as this was able to trigger a lowering of α-syn protein levels within the SN [52].

**Figure 7:**
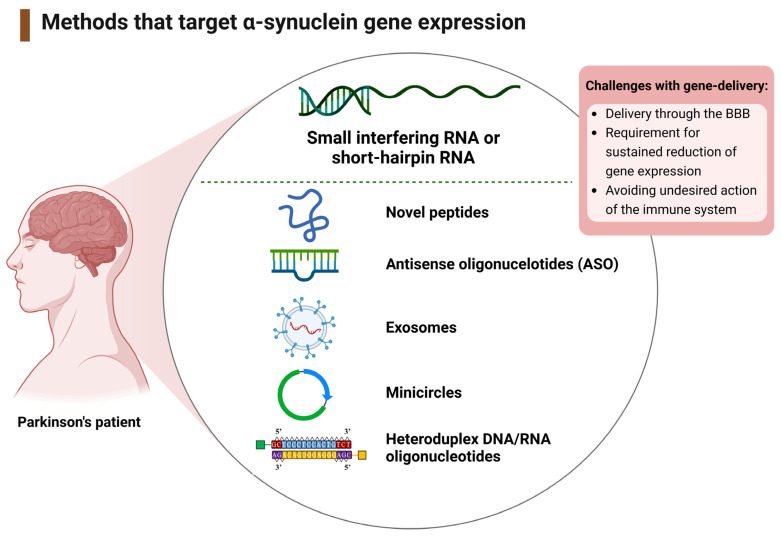
A summary of the main strategies for gene therapy interventions targeting α-syn.

A summary of the gene therapy delivery methods for the treatment of PD has been included in Figure 7.

### 4.3. Inhibition of α-Syn Aggregation

Inhibiting α-syn aggregation is an attractive target for combating the toxic aggregates that induce PD, and studies have focused on targeting thevaggregated forms of α-syn and/or promoting α-syn disaggregation [53,54,55,56,57,58,59,60,61,62]. 

#### 4.3.1. Small Molecules That Inhibit α-Syn Aggregation

Anle138b (2-(1,3-bnzodioxol-5-yl)-5-(3-bromophenyl)-1H-pyrazole), a DPP-derivate with high oral bioavailability and the ability to penetrate the BBB, has been utilized as an anti-aggregation inhibitor [53]. Anle138b successfully targeted α-syn oligomers but did not bind the α-syn monomer and may have an application to other proteinopathies [53]. Recently, a clinical trial was undertaken (randomized, double-blind, placebo-controlled Phase 1a) that provided evidence of the safety and tolerability of anle138b (NCT04208152) [78,79], indicative of the potential progression for an assessment of disease modifying effects.

Nanotheranostics, including the use of graphene quantum dots (GQDs), have been applied to neurodegenerative diseases such as PD [80]. GQDs were able to cross the BBB, inhibit fibril formation, and disaggregate mature fibrils without in vitro or in vivo toxicity [57]. 

Molecular tweezers have shown anti-aggregation properties by targeting positively charged residues in proteins that may undergo amyloidogenic changes [81]. The molecular tweezer CLR01 reduced α-syn aggregation and dissociated pre-aggregated α-syn [58], with its molecular size facilitating internalization by cells; a potentially advantageous property compared to some of the antibody-based therapies that require external epitopes. 

The small molecule *trans*-4′-acetyl-3′-tigloylkhellactone (racemic peucedanocoumarin IV (PCiv)) displayed anti-aggregation properties and was able to provide neuroprotection after oral administration to an α-synucleinopathy mouse model, but with a relatively low bioavailability of approximately 10% in rats [61].

The delivery of small molecules via red blood cell membrane (RBCm)-coated nanodecoys provides a means to reduce immune recognition and facilitate passage through the circulation and across the BBB. A rabies viral polypeptide (of 29 amino acids) modified by RBCm was used to encapsulate curcumin nanocrystals (RVG29-RBCm/Cur-NCs) [62]. The RVG29 peptide can specifically bind to acetylcholine receptors expressed in the BBB and neuronal cells and thereby facilitate the delivery of curcumin across the BBB [62]. This nanodecoy delivery of curcumin was able to inhibit α-syn aggregation, promote an increase in TH-positive cell number, and alleviate motor deficits in PD mice [62]. Furthermore, curcumin release ameliorated mitochondrial dysfunction and associated cellular redox stress [62]. 

#### 4.3.2. Enhancing α-Syn Degradation

An alternative approach to anti-α-syn therapy is to utilize factors that enhance α-syn turnover. Proteasomal, lysosomal, and autophagic pathways can all contribute to α-syn protein degradation [82]. Manipulation of the activity of the ubiquitin ligase, Nedd4, promoted α-syn degradation with an associated reduction of dopaminergic degeneration [54], underscoring the connection between α-syn degradation and synucleinopathy [82]. Hence, the therapeutic potential for modifying ubiquitination and protein turnover to limit α-syn accumulation through enhanced protein degradation. 

A prolyl oligopeptidase, PREP, can enhance the fibrilization of α-syn [83]. Hence, the administration of KYP-2047, a PREP inhibitor, to A30P transgenic mice decreased the levels of high molecular weight α-syn oligomers, presumably via KYP-2047-induced autophagy [55], although recent data has suggested that KYP-2047 treatment (initiated at the time of symptom onset) may not be protective to nigrostriatal dopaminergic neurons in a transgenic mouse PD model [84].

**Figure 8:**
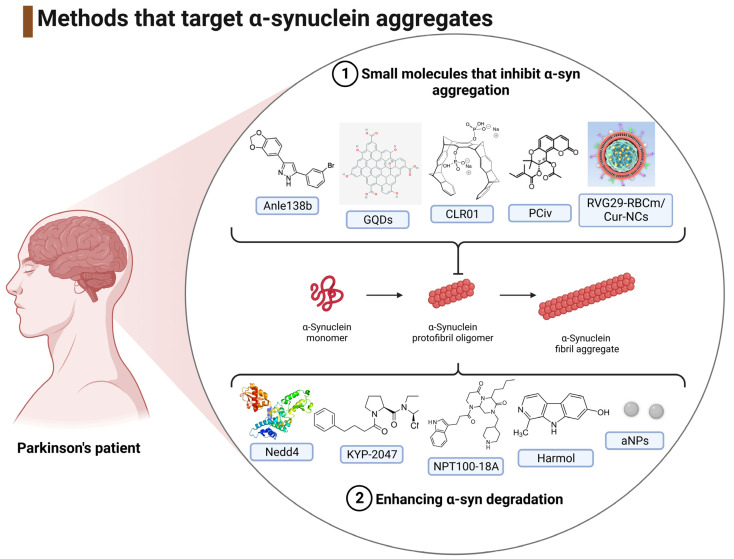
A summary of the strategies employed to reduce the level of α-syn aggregates.

An alternative approach utilized a de novo-developed compound, NPT100-18A, to displace α-syn from the membrane and reduce the formation of toxic oligomers [56]. Proteosome or autophagy inhibitors significantly reduced the effects of NPT100-18A, consistent with the mechanism that NPT100-18A enhances α-syn clearance via autophagic or proteasomal pathways [56]. Similarly, the employment of enhancers of autophagy (harmol) or lysosomal degradation (acidic nanoparticles) promoted the clearance of α-syn [60,61]. A summary of the anti-aggregation methods for the treatment of PD has been included in Figure 8.

### 4.4. Study Limitations

Although there are differences in relative volumes of brain areas between rodents and humans, in general, there are commonalities in the neuroarchitecture, with homologous functional networks and patterns of gene expression [85,86], hence the preponderance of rodent in vivo (PD) models. [85]. However, different models represent a source of variation between the preclinical studies. The use of α-syn-propagation models, transgenic animals, or chemical induction of PD via toxicants such as MPTP [87], variations between the species of α-syn studied, experimental time courses, and the extent of induction of toxic α-syn, could all impact study outcomes and influence the potential for clinical translation. 

Collectively, PD models that create α-syn fibrils are typically rapid in formation, much in contrast to the development of PD pathology and symptomology in humans, which can take decades to present. Therefore, although PD models have proved useful for the assessment of the efficacy of anti-α-syn therapies, the variety of neurotoxic effects cannot fully represent the spectrum of PD pathology observed in humans. Furthermore, it is still debated as to whether oligomeric or fibrillar species of α-syn are the most neurotoxic forms of the protein. Hence, studies discussed in this review that have assessed the efficacy of an intervention to preformed fibrils (PFFs) of α-syn might be less relevant in terms of their neuroprotective effects if oligomeric forms of the protein are the primary neurotoxic species in humans. Additionally, some pre-clinical studies have considered pSer129-α-syn as a marker of pathologically relevant aggregated α-syn. However, whether pSer129-α-syn represents a marker of disease status is controversial, and a recent study using post-mortem brain homogenates from PD patients has suggested that pS129–α-syn arises subsequent to protein aggregation and can inhibit further α-syn fibril formation and maybe, therefore, be generated as a neuroprotective response [88]. 

Furthermore, although the pre-clinical studies that have been discussed have similar and important disease-related endpoints such as decreased α-syn aggregation and increased dopaminergic neuron number and function, and for some studies, an assessment of the restoration of motor deficits was also undertaken, there is a limit to the ability to recapitulate the non-motor (and prodromal) symptoms present in PD, such as cognitive decline and psychiatric impairment. Hence, it will be useful to extend the range of animal models and, when possible, consider the assessment of therapeutic efficacy across multiple models, including those that are focused on prodromal (PD-specific premotor) symptoms [89]. 

The methodological study limitations include the use of a range of different animals (and specific rodent strains) as PD models extend to an inability to consider both female and male sexes, which may also influence the levels of α-syn expression and pharmaceutical outcomes. Lastly, administration techniques within preclinical models vary, and invasive administrative methods, such as stereotaxic interventions, are unlikely to translate to broad clinical trials. Routes of administration will also impact pharmacokinetic profiles.

## 5. Conclusions

**Figure 9:**
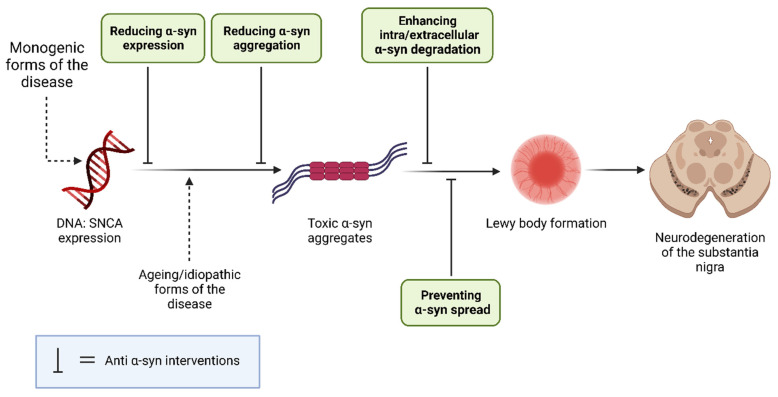
A summary of the intervention points of disease-modifying therapies targeting α-syn.

This systematic review has considered the effects of a range of methods that target α-syn aggregates using pre-clinical models and clinical trials for PD treatments, as summarized in Figure 9. Collectively, the in vivo animal studies have proven the efficacy of anti-α-syn therapeutics in reducing α-syn toxicity and pathology. Human clinical trials examining immunotherapies have displayed varying outcomes, with a few progressing into Phase II trials. Hence, the development and testing of a range of strategies to combat α-syn aggregation are cause for optimism, and it may transpire that a combinatory approach is most fruitful to halt disease progression. Furthermore, these novel disease-modifying therapies may be better exploited and more efficacious if used in combination with alternative therapeutic strategies (and mechanisms of action), such as the utilization of repurposed drugs [90,91,92]. 

However, a caveat remains for anti-α-syn therapy, and that is the need for an improved understanding of the physiological function of α-syn and how that becomes disrupted to evoke neurotoxicity and neuropathology since this will help direct therapeutic approaches. This will ensure that a substantive decrease of native α-syn (such as that through gene therapy) can be undertaken to an extent that it is not detrimental to its normal physiological function and importance in neurotransmission. 

Potential anti-α-syn mechanisms include suppression of α-syn expression and aggregation; enhancement of α-syn degradation; and prevention of α-syn spread.

## Figures and Tables

**Table 1:** Summary of mechanisms of action of immunological interventions.

Study	Agent	Type of Study	Treatment Strategy and Study Objectives
Sanchez-Guajardo et al., 2013 [35]	rAAv-α-syn	in vivo	Application of a neuroprotective vaccine to potentiate natural immune tolerance to α-syn.
Chatterjee et al., 2018 [36]	VH14*PEST and NbSyn87*PEST	in vivo	Utilization of human anti-α-syn nanobody constructs fused to a proteasome-targeting PEST sequence to enhance the clearance of target (α-syn) antigens.
Jankovic et al., 2018 (NCT02157714) and NCT03100149 [37,38]	PRX002	Phase I and II Clinical Trials	Assessment of the efficacy and safety of the humanized monoclonal antibody, prasinezumab, directed against the C-terminus of α-syn designed to prevent α-syn aggregation and its cell-to-cell transmission.
Ren et al., 2019 [39]	RVG-exosome aptamer	in vivo	RVG-exosome delivery of aptamers that recognize α-syn to reduce the formation of α-syn aggregates.
Schofield et al., 2019 [40]	MEDI1341	in vivo	Application of a high affinity antibody directed to the C-terminal of α-syn to sequester extracellular α-syn and attenuate α-syn spreading.
Volc et al., 2020 [41]	PD01A	Phase I Clinical Trial	Assessment of safety and tolerability of epitope mimetics of a C-terminal region of α-syn conjugated to a carrier protein to break immune tolerance and produce antigen-specific antibodies.
Poewe et al., 2021 [42]	PD03A	Phase I Clinical Trial	
Butler et al., 2022 [43]	AAV-EGFP-PFFNB2	in vivo	Utilization of an anti-α-syn nanobody (PFFNB2) fused with an AAV-EGFP to dissociate α-syn fibrils and limit α-syn spread.
Schmidhuber et al., 2022 [44]	WISIT vaccine	in vivo	Utilization of a DNA vaccine for multiple epitopes to reduce α-syn aggregation and propagation.
Roshanbin et al., 2022 [45]	RmAbSynO2-scFv8D3	in vivo	Utilization of a bispecific antibody to α-syn and the transferrin receptor to facilitate uptake across the BBB to target α-syn aggregates.

Abbreviations: EGFP, enhanced green fluorescent protein; PEST, proline, glutamic acid, serine, threonine; rAAV, recombinant adeno-associated virus; RVG, rabies viral glycoprotein; WISIT: Win the Skin Immune System Trick.

**Table 2:** Summary of the treatment strategies for SNCA gene therapy.

Study	Agent	Category of Gene Therapy	Treatment Strategy
Helmschrodt et al., 2017 [46]	PEI/SNCA-siRNA	Non-viral	Utilization of nanoparticle PEI to mediate the delivery of siRNA into the brains of mice to reduce expression of the SNCA gene and α-syn protein production.
Alarcón-Arís et al., 2018 [47]	IND-siRNA or IND-1233-ASO	Non-viral ASOs	Utilization of indatraline-conjugated ASO or siRNA to knockdown expression of the SNCA gene and α-syn protein production.
Izco et al., 2019 [48]	RVG-exosomes with anti-GFP shRNA-MCs	Viral delivery	Nanoparticle delivery of shRNA-MCs into the brain via RVG-exosomes to knockdown SNCA gene expression to limit the formation of α-syn aggregates.
Spencer et al., 2019 [49]	ApoB^11^	Non-viral	Conjugation of an 11 amino acid sequence of ApoB protein coupled to a 9 amino acid linker to deliver siRNA across the BBB to reduce α-syn levels.
Cole et al., 2021 [50]	ASO1	Non-viral ASOs	Utilization of ASOs targeting the SNCA gene to reduce the production of α-syn protein.
Jin et al., 2021 [51]	Tat-βsyn-degron	Non-viral	Utilization of Tat-βsyn-degron, a three-domain synthetic peptide designed to cross the BBB, bind to endogenous α-syn, and target it for proteasomal degradation.
Cao et al., 2022 [52]	HDOs	Non-viral	Utilization of HDOs to knockdown expression of the SNCA gene and α-syn protein production.

Abbreviations: ASO, antisense oligonucleotide; BBB, Blood-brain barrier; HDOs, heteroduplex oligonucleotides; IND; indatraline; PEI, polyethyleneimine; shRNA-MCs, short hairpin RNA-mini circles; siRNA; short interfering RNA.

**Table 3:** Summary of the mechanisms of action of studies that target α-syn aggregates.

Study	Agent	Intervention Category	Mechanism of Action
Wagner et al., 2013 [53]	Anle138b	Small molecule	Utilization of anle138b as an α-syn aggregation inhibitor derived from DPP.
Davies et al., 2014 [54]	Nedd4	Degradation enhancer	Utilization of Nedd4 as a ubiquitin ligase to target α-syn for lysosomal degradation.
Savolainen et al., 2014 [55]	KYP-2047	Degradation enhancer	Utilization of KYP-2047, a PREP inhibitor, to enhance clearance of α-syn via autophagy.
Wrasidlo et al., 2016 [56]	NPT100-18A	Small molecule	Utilization of NPT100-18A, a cyclic peptidomimetic derived from small peptides analogous to the 96–102 domain of α-syn, capable of displacing membrane-associated α-syn and reducing oligomer formation.
Kim et al., 2018 [57]	GQDs	Small molecule	Utilization of GQDs to inhibit fibrillization and enhance α-syn disaggregation.
Bengoa-Vergniory et al., 2020 [58]	CLR01	Small molecule	Utilization of CLR01 as a molecular tweezer to decrease α-syn aggregation.
Xu et al., 2022 [59]	Harmol	Degradation enhancer	Utilization of harmol to promote α-syn degradation by an autophagic-lysosomal pathway.
Arotcarena et al., 2022 [60]	aNPs	Degradation enhancer	Utilization of aNPs to promote α-syn degradation by enhanced lysosomal activity.
Kim et al., 2022 [61]	PCiv	Small molecule	Utilization of PCiv as an α-syn disaggregation agent.
Liu et al., 2022 [62]	RVG29-RBCm/Cur-NCs	Small molecule	Utilization of RVG29-RBCm/Cur-NCs a nanodecoy to act as an α-syn aggregation inhibitor able to cross the BBB.

Abbreviations: Anleb138b, 3-(1,3-benzodioxol-5-yl)-5-(3-bromophenyl)-1*H*-pyrazole; aNPs, acidic nanoparticles; DPP, di-phenyl-pyrazole; GQDs, graphene quantum dots; Nedd4, neural precursor cell expressed developmentally down-regulated protein 4; PCiv, peucedanocoumarin IV; PREP; prolyl oligopeptidase. RVG29-RBCm/Cur-NCs, Cur-NCs, rabies viral glycoprotein-29-red blood cell membrane/curcumin nanocrystals.

**Table 4:** Summary of biochemical and immunohistochemical analyses in immunotherapeutic interventions.

Study	Agent	Changes in α-Syn and TH in Intervention Groups (vs. PD Model)	Type of α-Syn Species Targeted	Level of Significance
Sanchez-Guajardo et al., 2013 [35]	rAAv-α-syn	↓α-syn aggregates TH levels similar to control	Aggregates	(*p* < 0.05) ND
Chatterjee et al., 2018 [36]	VH14/ NbSyn87*PEST	↓α-syn aggregates ↑TH-labelled cells	Aggregates	(*p* < 0.05) (*p* < 0.01) (VH14*PEST)
Jankovic et al., 2018 [37]	PRX002	↓Free-to-total serum α-syn	Aggregates	(*p* < 0.001)
Ren et al., 2019 [39]	RVG-exosome aptamer	↓α-syn	Fibrils aggregates	(*p* < 0.01)
Schofield et al., 2019 [40]	MEDI1341	↓α-syn	Oligomers	Hippocampal (*p* < 0.001) Neocortical (*p* < 0.001)
Volc et al., 2020 [41]	PD01A	↓α-syn	Oligomers	CSF (↓51% after 26 weeks at 75 µg), significance ND
Poewe et al., 2021 [42]	PD03A	ND	Oligomers	ND
Butler et al., 2022 [43]	AAV-EGFP-PFFNB2	↓α-syn aggregates (pS129)	Fibrils aggregates	Cortex (*p* < 0.001)
Schmidhuber et al., 2022 [44]	WISIT candidate type 1	↓α-syn aggregates (pS129)	Aggregates	(*p* < 0.05)
Roshanbin et al., 2022 [45]	RmAbSynO2-scFv8D3	α-syn (total) ↓α-syn oligomers	Oligomers aggregates	No change Cortex (*p* < 0.05) Midbrain (*p* < 0.005)

Abbreviations: ↑, denotes an increase; ↓, denotes decrease; CSF, cerebrospinal fluid; ND, not determined; EGFP, enhanced green fluorescent protein; PEST, proline, glutamic acid, serine, threonine; PFF, pre-formed fibril; pS129, phosphorylated (α-syn) at Serine129; rAAv, recombinant adeno-associated virus; RVG, rabies viral glycoprotein; TH, tyrosine hydroxylase; WISIT, Win the Skin Immune System Trick.

**Table 5:** Summary of biochemical and immunohistochemical analyses in gene therapy interventions.

Study	Agent	Changes in SNCA, α-Syn, TH, or Dopamine in Intervention Groups (vs. PD Model)	Location and Level of Significance for α-Syn, TH, or Dopamine
Helmschrodt et al., 2017 [46]	PEI/siRNA	↓mRNA and α-syn protein	Striatum (medial) (*p* = 0.018)
Alarcón-Arís et al., 2018 [47]	IND-siRNA or IND-1233-ASO	↓mRNA and α-syn protein ↑DA	SNc (*p* < 0.05) with IND-499-siRNA SNc (*p* < 0.05) with IND-1233-ASO CPu and medial PFC in response to veratridine (*p* < 0.05) with IND-1233-ASO
Izco et al., 2019 [48]	Exosomal RVG-anti-GFP shRNA-MCs	↓α-syn protein ↑TH-labelled cells	Midbrain, 90 day treatment (*p* = 0.033) (*p* = 0.028)
Spencer et al., 2019 [49]	ApoB^11^-siRNA	↓α-syn protein ↑TH-labelled cells	(*p* < 0.05) Striatum (*p* < 0.05)
Cole et al., 2021 [50]	ASO1	↓mRNA and α-syn aggregates ↑TH-labelled cells	SN (*p* < 0.001) for 100 and 300 µg dosing, (*p* < 0.0001) for 1000 µg dosing (*p* < 0.05)
Jin et al., 2021 [51]	Tat-βsyn-degron	↓α-syn ↑TH-labelled cells	SN (*p* < 0.01) SN (*p* < 0.05)
Cao et al., 2022 [52]	HDO	↓α-syn ↑TH-labelled cells	SN (*p* < 0.05) SN (*p* < 0.01)

Abbreviations: **↑**, denotes an increase; **↓,** denotes decrease; ASO, antisense oligonucleotide; CPu, Caudate putamen; DA, dopamine; HDOs, heteroduplex oligonucleotides; IND, indatraline; PEI, polyethyleneimine; PFC, Prefrontal cortex; RVG, rabies viral glycoprotein; shRNA-MCs short hairpin RNA-mini circles; siRNA, short interfering RNA; SNc, substantia nigra pars compacta; TH, tyrosine hydroxylase.

**Table 6:** Biochemical and immunohistochemical outcomes of agents reducing α-syn aggregates.

Study	Agent	Changes in α-Syn, Dopamine, and TH in Intervention Groups (vs. PD Model)	Type of α-Syn Species Targeted	Level of Significance
Wagner et al., (2013) [53]	Anle138b	↓α-syn	Oligomeric	(*p* < 0.001)
Davies et al., 2014 [54]	Nedd4	↓α-syn ↑TH-labelled cells	Oligomeric	(*p* = 0.022) (*p* < 0.05)
Savolainen et al., 2014 [55]	KYP-2047	↓α-syn ↑DA TH	Oligomeric	28-d treatment: (*p* = 0.0028) 28-d treatment: (*p* = 0.01) NS
Wrasidlo et al., 2016 [56]	NPT100-18A	↓α-syn	Oligomeric	(*p* < 0.05)
Kim et al., 2018 [57]	GQDs	↓α-syn ↑TH-labelled cells	Fibrillar	(*p* < 0.001) (*p* = 0.0156)
Bengoa-Vergniory et al., 2020 [58]	CLR01	↓α-syn ↑TH-labelled cells	Oligomeric	(*p* = 0.0286) (*p* = 0.0177)
Xu et al., 2022 [59]	Harmol	↓α-syn	Total	SN and PFC (*p* < 0.05)
Arotcarena et al., 2022 [60]	aNPs	α-syn (pSer129) ↑TH-labelled cells	Aggregates	NS (total/proteinase K-resistant), pSer129 (*p* < 0.05) (*p* < 0.05)
Kim et al., 2022 [61]	PCiv	↓α-syn (pSer129) ↑TH-labelled cells	Aggregates	SN (*p* < 0.01) SN (*p* < 0.001)
Liu et al., 2022 [62]	RVG29-RBCm/Cur-NCs	↓α-syn ↑DA ↑TH	Total	Midbrain and striatum (*p* < 0.01) (*p* < 0.001) Midbrain and striatum (*p* < 0.01)

Abbreviations: ↑, denotes increase; ↓, denotes decrease; Anleb138b, 3-(1,3-benzodioxol-5-yl)-5-(3-bromophenyl)-1*H*-pyrazole; aNPs, acidic nanoparticles; GQDs, graphene quantum dots; PCiv, peucedanocoumarin IV; PFC, prefrontal cortex; pSer129, phosphorylated α-syn at Ser129; Nedd4, neuronally expressed developmentally down-regulated gene 4; NS, not significant; RVG29-RBCm/Cur-NCs, rabies viral polypeptide-29-red blood cell membrane/curcumin nanocrystals; SN, substantia nigra; TH, tyrosine hydroxylase.

## Data Availability

Data associated with results table generation is available on request from the last author.

## Supplementary Materials

The following supporting information can be downloaded at: https://www.mdpi.com/article/10.3390/ijms241311022/s1.
