# Supplementary material for: Are Therapies That Target α-Synuclein Effective at Halting Parkinson’s Disease Progression? A Systematic Review
Source: Int J Mol Sci. 2023 Jul 3;24(13):11022. doi: 10.3390/ijms241311022 (PMC10341763; doi:10.3390/ijms241311022)
Supplement: Supplementary file 1 [file ijms-24-11022-s001.zip › ijms-2464752-supplementary.pdf]

Supplementary Data S1

An electronic literature database search was conducted using: OVID (Medline and Embase), PubMed, Web of Science Core Collection and Cochrane. The following search terms were utilised to retrieve relevant publications regarding in vivo studies and human trials exploring anti-α-syn therapies. Boolean operators were used to conjugate search terms and field tags such as topic (TS) and title (TI) were used to explore a range of key terms.

OVID (Medline and Embase) search terms:

1. Parkinson's Disease or Parkinson*
2. target* alpha-syn* OR anti-alpha-syn* OR decreas* alpha-syn* OR prevent* alpha-syn
3. *In vivo* OR pre-clinical OR human trial
4. Human OR animal OR primate OR monkey OR rodent OR mouse OR mice OR rat
5. #1 AND #2 AND #3 AND #4

PubMed search:

(Parkinson's Disease OR Parkinson*) AND (anti-alpha-syn* therap* OR target* alpha-syn* therap*)

Web of Science Core Collection:

1. TS (topic) = Parkinson's Disease OR Parkinson*
2. T1 (title) = Parkinson's Disease OR Parkinson*
3. #1 OR #2
4. TS (anti-alpha-syn* therap* OR target* alpha-syn*)
5. T1 (anti-alpha-syn* therap* OR target* alpha-syn*)
6. #4 OR #5
7. TS (Human OR animal OR primate OR monkey OR rodent OR mouse OR mice OR rat)
8. T1 (Human OR animal OR primate OR monkey OR rodent OR mouse OR mice OR rat)
9. #7 OR #8
10. #3 AND #6 AND #9

Cochrane:

1. Parkinson's Disease or Parkinson*
2. target* alpha-syn* OR anti-alpha-syn* OR decreas* alpha-syn* OR prevent* alpha-syn

3. Human OR animal OR primate OR monkey OR rodent OR mouse OR mice OR rat

4. In vivo OR pre-clinical OR human trial

5. #1 AND #2 AND #3 AND #4
